# Regulation of inflammation during wound healing: the function of mesenchymal stem cells and strategies for therapeutic enhancement

**DOI:** 10.3389/fphar.2024.1345779

**Published:** 2024-02-15

**Authors:** Mingnan Gao, Han Guo, Xuan Dong, Zimao Wang, Zheng Yang, Qiaoli Shang, Qiying Wang

**Affiliations:** Department of Plastic Surgery, First Affiliated Hospital of Zhengzhou University, Zhengzhou, Henan, China

**Keywords:** skin, wound healing, MSCs, inflammation, immune response, pre-implant optimization

## Abstract

A wound takes a long time to heal and involves several steps. Following tissue injury, inflammation is the primary cause of tissue regeneration and repair processes. As a result, the pathophysiological processes involving skin damage, healing, and remodeling depend critically on the control of inflammation. The fact that it is a feasible target for improving the prognosis of wound healing has lately become clear. Mesenchymal stem cells (MSCs) are an innovative and effective therapeutic option for wound healing due to their immunomodulatory and paracrine properties. By controlling the inflammatory milieu of wounds through immunomodulation, transplanted MSCs have been shown to speed up the healing process. In addition to other immunomodulatory mechanisms, including handling neutrophil activity and modifying macrophage polarization, there may be modifications to the activation of T cells, natural killer (NK) cells, and dendritic cells (DCs). Furthermore, several studies have shown that pretreating MSCs improves their ability to modulate immunity. In this review, we summarize the existing knowledge about how MSCs influence local inflammation in wounds by influencing immunity to facilitate the healing process. We also provide an overview of MSCs optimizing techniques when used to treat wounds.

## Introduction

The biggest multipurpose organ in the human body, the skin, acts as a barrier to keep out harmful materials and creatures from the external environment, such as toxins and physical assaults (Yang J. et al., 2020). A wound is a physical harm that happens when the skin deforms due to damage to the skin’s internal and exterior components (Ayavoo et al., 2021). Wound healing can be impacted by several variables, including infection, foreign substances, necrosis, and comorbidities, even though the skin can restore itself to its normal condition and function (Falanga, 2005; Han and Ceilley, 2017). Prolonged wound closure raises the possibility of bacterial colonization, invasion, and even sepsis, all of which can be fatal for patients (Lemsanni et al., 2021; Soh, 2021).

Traditional wound treatment options comprise the application of antibiotics, various dressings, debridement, laser therapy, biological scaffolds, topical administration of specific growth factors, skin/flap grafting, gene therapy, and negative-pressure suction. These techniques have certain drawbacks, such as the lengthy treatment duration, high cost, and significant psychological and financial stress placed on patients (Qin et al., 2023). The initiation of tissue regeneration and repair is commonly attributed to the activation of the inflammatory response (Zhu et al., 2023). During the whole healing process, the initial stage is the inflammatory reaction. Inflammation is caused by the invasion of an area by inflammatory cells, which activates both the innate and adaptive immune systems (Gentek and Hoeffel, 2017; Rieckmann et al., 2019). Excessive inflammatory reactions can lead to severe pathological changes, including scarring and impeded wound healing. In contrast, well-controlled inflammation often promotes wound healing (Xu et al., 2012; Ogawa, 2017; Wang Z.-C. et al., 2020; Yu et al., 2021; Zhang et al., 2021). Thus, it is critical for wound healing to identify a treatment regimen that effectively modulates immune-related cells and cytokines at the site of damage to reduce inflammation at the appropriate time point (Hong et al., 2023).

Recent years have seen a notable advancement in the field of study on mesenchymal stem cells’ (MSCs) ability to promote wound healing. A subset of pluripotent stem cells known as MSCs was found in the bone marrow stroma during the 1960s. When transplanted into ectopic areas, these cells may be able to generate new bone (Friedenstein et al., 1970; Bianco et al., 2013). MSCs are thought to be the best seed cells for tissue engineering, according to recent research (Tsiapalis and O’Driscoll, 2020; Khayambashi et al., 2021). They may be extracted from a variety of tissues, such as the skin, bone marrow, adipose, umbilical cord, and dental pulp. In tissue injury, it not only has an extremely strong self-proliferation, multidirectional differentiation ability, and powerful secretion ability, but more importantly, it has an immunomodulatory function that can form a favorable inflammatory microenvironment at the site of tissue injury, release growth factors, and activate endogenous tissue repair (Wang et al., 2014; Wang et al., 2022). Existing evidence suggests that MSCs-based therapies can control and reduce inflammation but not replace damaged cells. MSCs have been used, for example, to alter organ inflammation at different tissue locations in the early stages of treating acute and chronic liver damage. For COVID-19, intravenous infusion of MSCs has also been suggested as a treatment to suppress inflammatory storms in the lungs (Verkhratsky et al., 2020). As a result, the use of MSCs in regenerative medicine must highlight both their limited ability to differentiate and their role in immunomodulation, which entails establishing a supportive immune environment and secreting growth factors to promote endogenous tissue healing (Wang et al., 2014).

However, the relationship between MSCs and inflammation is complex. It has been suggested that the immune regulation of MSCs is not intrinsic but is triggered by inflammation and is dynamically influenced by the type and amount of inflammatory cytokines (Ren et al., 2008; Wang et al., 2014). As an alternative, MSCs might respond to changes in tissue pathophysiological signals in an active manner. This would mobilize many components of the tissue immune microenvironment to achieve inflammation control, which would then aid in tissue regeneration and repair (Wang et al., 2014; Gao et al., 2021; Soliman et al., 2021). Currently, in vitro-amplified MSCs are a hotspot for therapeutic research, but there are still some outstanding problems. It has been discovered that in vitro-amplified MSCs are quickly identified and eliminated by the host’s immune system upon exogenous delivery. In addition, when MSCs are administered to some tissues with low inflammatory status, changes in their inflammatory microenvironment may lead to unstable efficacy of MSCs. These problems are considerable obstacles to its application. Therefore, knowing how to treat in vitro-expanded MSCs before transplantation is significant to improve their clinical efficacy. To increase the therapeutic effects of MSCs, many researchers have recently investigated diverse bioengineering methodologies and approaches for optimizing and pre-treating MSCs (Wang et al., 2022).

We have reviewed recent studies on the mechanisms of the role of MSCs in wound healing. In particular, we focused on the immunomodulatory role mediated by MSCs during the inflammatory phase. This article also presents a review of studies that strive to increase the therapeutic potential of MSCs and analyzes their implications for optimizing MSCs usage for tissue regeneration in clinical settings.

## Inflammatory mechanisms in the wound healing process

Dynamic and multifaceted interactions between a variety of cells, growth factors, cytokines, chemokines, and other chemicals are necessary for wound healing. Three stages are involved in the recovery of wounds: proliferative, remodeling, and hemostatic/inflammatory (Gurtner et al., 2008; Hong et al., 2023). These stages occur sequentially, although they also overlap. To initiate tissue repair and regeneration, the hemostatic/inflammatory phase is essential (Zhu et al., 2023). The sudden creation of a platelet-fibrin clot at the wound site is known as hemostasis, and it is triggered by severe skin damage. It involves almost immediate platelet aggregation and vasoconstriction (Clark, 2003; Zeng and Liu, 2021). This clot stops blood flow, lessens subsequent blood loss, and acts as a scaffold for the migration of immune and resident skin cells (Reish and Eriksson, 2008). Therefore, the histamine, bradykinin, and leukotrienes produced by nearby mast cells draw immune cells and widen blood vessels (Hong et al., 2023). Neutrophils are activated after skin injury as “first responders” by recruiting “find me” signals from the blood (Zhu et al., 2023). Several signals are produced by the wounded or infected area, including lipid mediators, hydrogen peroxide, damage-associated molecular patterns (DAMPs), calcium waves, and chemokines (Su and Richmond, 2015). Many tactics are used by activated neutrophils to degrade pathogens by proteolytic hydrolysis and oxidative bursts of microbicidal reactive oxygen species (ROS) released. This process is known as neutrophil extracellular traps (NETs) formation (Clark et al., 2007; Kolaczkowska and Kubes, 2013; Lood et al., 2016). Activated neutrophils also use phagocytosis and expel DNA decorated with particles from proteolytic hydrolysis to immobilize and eventually kill pathogens. Macrocytizing macrophages phagocytize neutrophils that have undergone extensive apoptosis in the final stages of inflammation. Inflammatory monocytes are drawn from the peripheral circulation 48–96 h after injury, and when they get to the site of damage, they change into macrophages (Geissmann et al., 2010). Macrophages (also called M1 type) are essential during the early stages of wound healing because they help promote angiogenesis and fibroplasia, synthesize nitric oxide (NO), and move the wound from an inflammatory to a proliferative phase. By producing pro-inflammatory cytokines, including tumor necrosis factor alpha (TNF-*α*), interleukin-1*β* (IL-1*β*), and IL-6, they can also fight off infection. Reducing inflammation and restoring tissue homeostasis is made possible by macrophages’ ability to develop anti-inflammatory qualities and support tissue healing. The anti-inflammatory type M2 phenotype is developed as a result of this change. In response to IL-4/IL-13 or apoptotic neutrophils, type M2 macrophages produce platelet-derived growth factor (PDGF), transforming growth factor-*β* (TGF-*β*), insulin-like growth factor-1 (IGF-1), and vascular endothelial growth factor (VEGF), which encourages the proliferation of keratinocytes and fibroblasts (Russell et al., 2019). Dendritic cells (DCs) provide antigens, which stimulate T-cell responses. Helper T-cells 1 (Th1), which have proinflammatory effects during the acute inflammatory phase of the wound, are primarily driven by interferon-*γ* (IFN-*γ*). The T cells get more and more polarized throughout the inflammatory phase, which eventually turns into a Th2 response. These cells produce IL-4, IL-5, IL-10, and IL-13, which are anti-inflammatory and also have significant pro-fibrotic effects (Wallace et al., 1994; Ong et al., 1999). Th17 cells induce an immune response that overlaps with Th1 and Th2 immune responses, promoting follicular neogenesis and pro-inflammatory effects through neutrophil recruitment and wound re-epithelialization (Zhu et al., 2023). Moreover, it has been found that regulatory T cells (Tregs) promote tissue homeostasis and regeneration after tissue injury by reducing excessive inflammation (Zhu et al., 2023) Figure 1.

**FIGURE 1:**
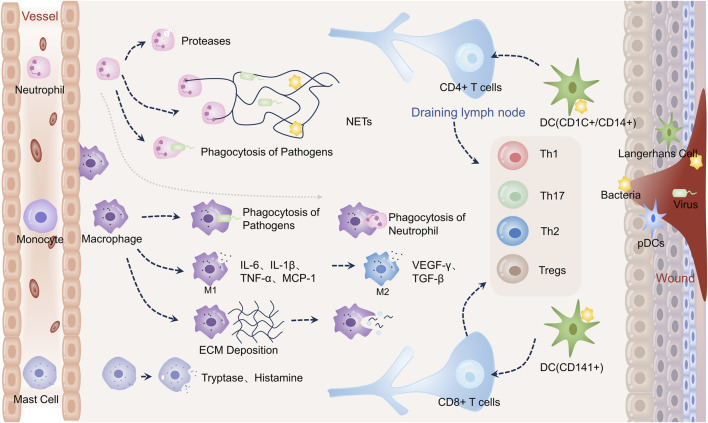
Immune cells in wound healing. Neutrophils directly phagocytose pathogens during wound healing, release proteases for hydrolysis, and create neutrophil extracellular traps (NETs) to engulf them–activation of monocytes into macrophages. Early inflammation causes pathogens to be phagocytosed by macrophages. In order to combat infection, M1-type macrophages emit proinflammatory molecules such as IL-6, IL-1*β*, and TNF-*α*. They also release monocyte chemokine-1 (MCP-1) to draw in more monocytes. Macrophages phagocytose apoptotic neutrophils later in the inflammatory process. M2-type macrophages stimulate angiogenesis and extracellular matrix deposition by releasing VEGF-*γ* and TGF-*β*. Macrophages phagocytose extracellular matrix and remnants of cells during remodeling. Certain DCs activate T cells, which subsequently differentiate into CD4^+^ and CD8^+^ T cells, respectively. Additionally, plasmacytoid DCs (PDC) in injured skin are triggered to generate interferon-*α* (IFN-*α*), a critical component of the acute inflammatory response. Tryptase and histamine are produced by mast cells.

## The control of inflammation impacts the prognosis of wound healing

While an overabundance of inflammation leads to scarring, delayed wound healing, and perhaps severe pathological changes, a moderate amount of inflammation encourages wound repair (Xu et al., 2012; Ogawa, 2017; Wang Z.-C. et al., 2020; Yu et al., 2021; Zhang et al., 2021). Inflammation is a necessary component in the healing of wounds. The length and severity of the inflammatory response affect how well the lesion heals. Consequently, in order to facilitate wound healing, it is crucial to preserve a suitable and timely balance between the inflammatory and reparative phases (Hong et al., 2023). Targeting pathways that either lower inflammation or trigger repair has been the focus of intense research over the past 30 years in an effort to enhance the prognosis of wound healing/. Examples of these efforts include the use of curcumin (Akbik et al., 2014), neurotensin (NT) (Liu et al., 2019), photodynamic therapy (PDT) (Yang T. et al., 2020), silver nanoparticles (AgNPs) (Choudhury et al., 2020), and others.

Curcumin’s ability to lower inflammation is supported by the majority of research (Akbik et al., 2014). By inhibiting the synthesis of TNF-*α* and IL-1 as well as the activation of the nuclear factor *κ* light chain enhancer (NF- *κ* B) in activated B-cells, curcumin controls inflammation in wounds (Joe et al., 2004). As the primary source of inflammation during wound healing activity, Gopinath et al. (2004) discovered that curcumin had a scavenging impact on ROS. Unfortunately, curcumin’s quick metabolism, low solubility, high photosensitivity, and low bioavailability restrict its medicinal usefulness (Akbik et al., 2014).

NT is also a critical immunomodulator that modulates cellular functions in innate and adaptive immunity (Evers et al., 1994; Ramez et al., 2001; Bernatchez et al., 2013). Moura et al. (2014) showed that the expression of IL-1*β* was reduced in mice treated with NT and NT-loaded collagen, which promoted wound healing. Additional research is required to address this issue as NT therapy alone has little therapeutic potential, as does NT-collagen loading treatment. However, it dramatically accelerates wound healing and leaves more noticeable scars once recovery is complete (Moura et al. (2014).

PDT has drawn a lot of interest as a wound therapy method in recent years. Research has indicated that PDT creates pro-inflammatory cytokines such as IL-1, lipids, and a local acute inflammatory response (Gollnick et al., 2006; Brackett and Gollnick, 2011; Anzengruber et al., 2015). It also dramatically influences neutrophil activation. However, 5-aminolevulinic acid (ALA) -PDT treatment was effective in diminishing inflammatory infiltration with relatively low expression levels of proinflammatory cytokines, such as TNF-*α*, IL-1*β*, and IL-23, according to Yang et al. (2021). In the mid-to-late phases of the wound-healing process, PDT therapy can decrease chronic inflammation and increase acute inflammation early in the healing phase (Yang T. et al., 2020). This indicates that PDT therapy can regulate inflammation and facilitate wound healing. However, PDT has some limitations, including pain and discomfort during treatment, long treatment duration, and instability of treatment results (Cottrell et al., 2008; Syed Azhar et al., 2018; Kaw et al., 2020), and its limited optical depth limits its application in deep tissue therapy (Li and Lin, 2022).

The anti-inflammatory activity of AgNPs has also been demonstrated in skin wounds (Tian et al., 2007; Nadworny et al., 2008; Liu X. et al., 2014; Wasef et al., 2020). It has been found that increased expression of type I collagen alpha 1 chain (Col1A1) suppressed inflammatory cytokines (IL-1*α* and IL-6), and inflammation was maintained at low equilibrium levels after treatment with AgNPs, effectively facilitating the healing process and decreasing the risk of chronicity in wounds (Shahverdi et al., 2007). The keratinocyte growth factor/p38 mitogen-activated protein kinase (KGF-2/p38MAPK) signaling pathway, which AgNPs mediate, controls the inflammatory response by promoting the production of early pro-inflammatory cytokines while inhibiting the early phase of the skin wound anti-inflammatory cytokine, IL-10, following injury. Zhang et al. (2020) showed that while early inflammation is crucial, prolonged inflammation is harmful to wound healing. In fact, giving mice a little late injection of AgNPs to reduce inflammation actually aided in their ability to repair wounds. However, in wounds, cell types are diverse, and the microenvironment is spatially heterogeneous, so targeting specific inflammatory factors or signaling pathways does not achieve great effectiveness (Gurtner et al., 2008; Forrester et al., 2009; Wang et al., 2022).

Therefore, the modulation of the inflammatory microenvironment is crucial in promoting wound healing. MSCs are gradually becoming the focus of promoting wound repair by modulating the inflammatory microenvironment of wounds from multiple mechanisms.

## MSCs enhance wound healing by regulating inflammatory response via immune modulation

Previous research has indicated that MSCs’ primary capacity to facilitate wound healing may stem from their secretion of pro-regenerative cytokines (Garg et al., 2014). As per the latest research, MSCs could suppress immune responses in the presence of enriched inflammatory cytokines, including infection, trauma, or immune-mediated diseases. These immunomodulatory properties were discovered in preclinical and clinical trials (Margiana et al., 2022). Therefore, when it comes to the use of in vitro-expanded MSCs in regenerative medicine, we shouldn’t overemphasize their limited differentiation potential (“cell replacement”). Instead, we should concentrate on a range of responses that MSCs have to injury signals, such as migration, immunomodulation, growth factor release, tissue remodeling, etc. Growth factors are released to start endogenous tissue healing (“cellular empowerment”) and to create an environment that is favorable for the immune system (Wang et al., 2014). Specifically, MSCs exert their inflammatory modulatory effects mainly relying on direct cell-to-cell communication and paracrine secretion (Keshtkar et al., 2018). Almost all immune cells, including mast cells, natural killer (NK) cells, DCs, T lymphocytes, and so on, can interact with and change the activity of MSCs in order to restore immunological homeostasis and prevent the immune response from spiraling out of control (Wang et al., 2014; Shi et al., 2018a; Han et al., 2022). Paracrine effects are thought to be the primary mechanism of their immunomodulation (Qiu et al., 2018; Qin et al., 2023). By releasing a range of growth factors, inflammatory modulators, and extracellular vesicles (e.g., exosomes (Exos)), the local inflammatory response can be regulated by MSCs after they enter an inflammatory environment (Shi et al., 2018b). MSCs positively impact wound healing, which is supported by their capacity to control the inflammatory response to wounds. The number of inflammatory cells and pro-inflammatory cytokines was dramatically reduced by human umbilical cord-derived MSCs (e.g., IL-1, IL-6, and TNF-*α*) in a rat model of severe burns, according to Liu L. et al. (2014). Crucially, these wounds also showed the higher speed of healing and shorter recovery periods (Liu L. et al., 2014). Further studies have shown that Exos in the MSCs secretome are increasingly recognized as critical intercellular mediators for transmitting biological signals rather than wasted products initially thought to be processed by cells. Numerous studies have demonstrated that Exos produced from MSCs actively participate in every step of wound healing (Qin et al., 2023) Figure 2

**FIGURE 2:**
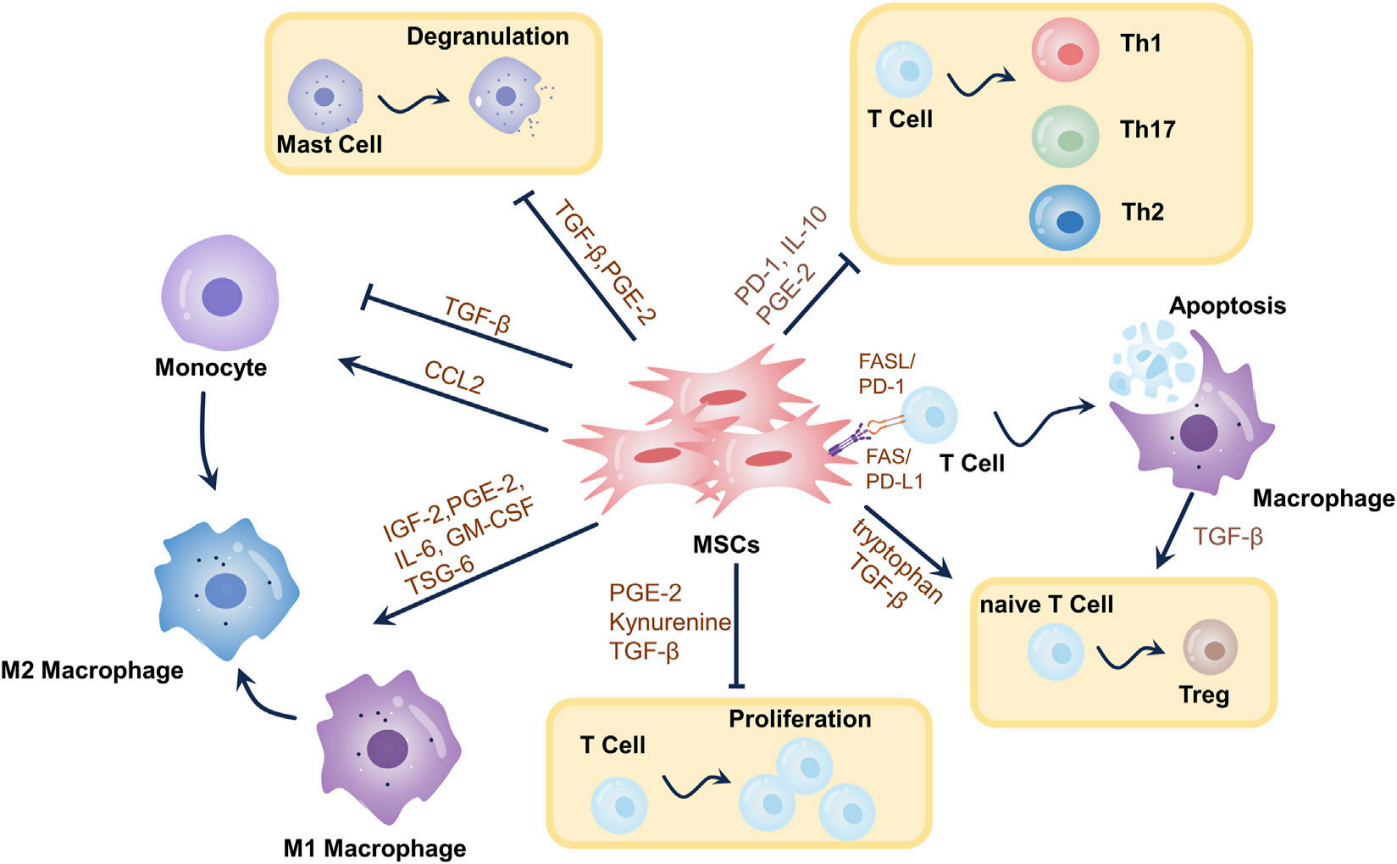
Mechanisms of immunomodulatory effects of MSCs in wound healing. Through the synthesis of metabolites, cytokines, growth factors, chemokines, exosomes, etc., as well as T-cell death-mediated immunomodulation, MSCs elicit immunomodulatory effects. MSCs can stimulate the differentiation of primitive CD4^+^ T cells into Tregs, restrict T-cell proliferation, and teach macrophages to adopt an immunosuppressive phenotype (M2-type). MSCs induce T-cell death, which triggers TGF-*β* release from macrophages, leading to Tregs differentiation and immunological tolerance. MSCs induce increased T-cell differentiation towards Th2 and decreased differentiation towards Th1 and Th17. MSCs promote mast cell degranulation. These mechanisms modulate the local inflammatory environment of the wound, thereby promoting wound healing.

### MSCs regulate neutrophil activity

An increasing amount of evidence points to the possibility that excessive neutrophil infiltration, which results in an overabundance of NETs, may damage the structure of the wound, hinder angiogenesis in the vicinity of the injury, and alter the quantity or quality of cells participating in wound repair, all of which might delay the healing process (Zhu et al., 2021). The neutrophils’ departure shows a decrease in inflammation. On the other hand, chronic wounds include diabetic foot ulcers, pressure sores, and venous leg ulcers, which are caused by persistent inflammation that is brought on by neutrophil persistence (Chen and Rogers, 2007). It was found that treatment with MSCs or MSCs conditioned medium (CM) significantly reduced the number of infiltrating neutrophils and alleviated progression in an acute injury mouse model (Ionescu et al., 2012; Wang et al., 2018). Interestingly, however, lipopolysaccharide (LPS) -activated MSCs present different regulatory effects on neutrophils. *In vitro* experiments by Brandau et al. (2014) showed enhanced neutrophil phagocytosis and increased survival after CM exposure to LPS-activated MSCs. Munir et al. (2020) found that injecting LPS-induced MSCs into mouse wounds significantly accelerated wound healing compared to uninduced MSCs. This was due to increased neutrophil granulocyte activation, NET formation, and ROS production, resulting in improved wound healing in alloplastic wounds. During the later phases, a large number of apoptotic neutrophils were taken up by macrophages, and there was an increase in TGF-*β* release. In turn, this promoted neovascularization and sped up the healing process overall by stimulating myofibroblast differentiation and wound contraction. However, in the repair of specialized wounds, such as diabetic wounds, excessive NETs formation is detrimental to wound healing. Chu et al. (2023) found that Exos derived from hypoxia-pretreated MSCs could promote wound healing by blocking excessive NETs formation through the transfer of miR-17-5p to target the Toll-like receptor-4 (TLR-4)/ROS/MAPK pathway. This shows that the role of neutrophils varies from one wound to another and from one period of time to another. Therefore, when applying the property of immunomodulation of MSCs for wound treatment, it is necessary to consider different characteristics of wounds and other periods of inflammation to understand more thoroughly the need for neutrophil activity in wounds and to use MSCs to regulate neutrophils more precisely. Still lacking, however, is a thorough understanding of how MSCs provide adaptive responses to modify neutrophil activity and activation state following tissue damage.

### MSCs regulate macrophage polarization

The primary way that MSCs influence macrophages is by controlling the polarization of these cells. Zhang et al. (2010) observed that following co-culturing with MSCs, macrophages exhibited an anti-inflammatory M2 phenotype, indicated by elevated levels of the anti-inflammatory cytokine IL-10 (Fairweather and Cihakova, 2009; Kim and Hematti, 2009; Martinez et al., 2009), a reduction in the pro-inflammatory cytokine TNF-*α*, and an increase in the expression of CD206, a recognized marker of M2 macrophages. Most importantly, injection of MSCs also led to a decrease in local inflammation, an increase in angiogenesis, and a notable speeding up of the healing process of the mouse wound, indicating that MSCs can support the M2 macrophage phenotype, which helps with cutaneous wound healing. da Costa Manso et al. (2023) found that skin wound healing was accelerated in diabetic mice receiving biotherapeutics containing MSCs and detected increases in TGF-*β* gene expression and M2 macrophage-related markers. These results confirmed findings from other studies suggesting that M2-type macrophages produced mediators necessary for tissue remodeling and inflammation reduction, thereby promoting wound repair (Tiemessen et al., 2007; Fairweather and Cihakova, 2009). Research has demonstrated that MSCs can release PGE2, which changes the phenotype of macrophages from polarized M1-type macrophages to M2-type (Spaggiari et al., 2009; Chen et al., 2018; Ko et al., 2020). To control macrophage polarization, MSCs release large amounts of the anti-inflammatory cytokine IL-6 (Philipp et al., 2018). Granulocyte-macrophage colony-stimulating factor (GM-CSF) and IL-6 generated by MSCs have been shown to significantly accelerate wound healing by causing macrophages to undergo M2 polarization (Spaggiari et al., 2009; Zhang et al., 2010). In a mouse model of injury, Zhao et al. demonstrated that MSCs-produced TSG6 induced polarization from pro-inflammatory to anti-inflammatory M1 phenotypes and sped up tissue regeneration by inhibiting the NF-*κ*B pathway (Zhao et al., 2021). Interestingly, via binding to CD44, TSG6 can also prevent macrophage migration into areas of inflammation and damage (Day and Milner, 2019). Furthermore, it has been discovered that human MSCs cultivated in hypoxia could produce IGF2, which acts on monocytes during their maturation from monocyte to macrophage, giving them long-lasting anti-inflammatory qualities rather than directly influencing Tregs differentiation or the polarization of mature macrophages. This characteristic shows that MSCs may continually repair aberrant immunological responses and restore immune homeostasis since it lasts long after they mature into macrophages and even after stimulation with LPS or IFN-*γ* (Du et al., 2019; Wang X. et al., 2020). It is commonly recognized that wound healing requires reestablishing local immunological homeostasis and rectifying aberrant immune responses. A growing body of research suggested that MSCs-derived apoptotic vesicles or exosomes might also accelerate the healing of cutaneous wounds by inducing macrophage polarization toward the M2 phenotype or by changing M2 polarization (He et al., 2019; Luo et al., 2021; Geng et al., 2022). Exos derived from melatonin-stimulated MSCs have been used to target the PTEN/AKT pathway, modulate macrophage M2 polarization, and increase IL-10 and arginase-1 (Arg-1) expression in order to enhance diabetic wound healing (Liu et al., 2020). According to one study, systemic treatment of MSCs produced from bone marrow could cause macrophages to become more polarized toward the M2 phenotype. It facilitates their migration to the site of the lesion. Following investigations have demonstrated that miR-223, which is produced from bone marrow MSC-Exos, targets pknox1 to control macrophage polarization and facilitate the healing of wounds (He et al., 2019). In conclusion, MSCs can control macrophage polarization via a variety of mechanisms, which is critical for wound healing and restoration Table 1.

**TABLE 1:** Factors of MSCs in the regulation of macrophage polarization.

Factors	Effectiveness	Mechanisms	Reference
TSG6	Induce M2 macrophages polarization	Suppress NF- *κ* B pathway activation	Zhao et al. (2021)
	Inhibit macrophages migration	Bind to CD44	Day and Milner (2019)
PGE2	Convert macrophages into regenerative phenotype	Not mentioned	Ko et al. (2020)
IL-6	Promote the generation of a regulatory M2b phenotype	Not mentioned	Philipp et al. (2018)
GM-CSF	Promote macrophage polarization to M2 type	Not mentioned	Zhang et al. (2010)
IGF2	Endow monocytes with permanent anti-inflammatory properties	Activate IGF2R and preprogram mature macrophages for oxidative phosphorylation (OXPHOS)	Du et al. (2019); Wang et al. (2020a)
miR-223	Promote macrophage polarization to M2 type	Targeted regulate the pknox1 gene	He et al. (2019)

### MSCs regulate T-cell activity

The final immune cells that penetrate the wound are T cells, which are crucial to the skin’s adaptive immunological response. T cells are drawn to chemokines generated within the wound, including chemokine C-C motif ligand-9 (CCL9) and CCL10 (Balaji et al., 2015). According to research, MSCs primarily control T-cell differentiation and proliferation by inhibiting T-cell proliferation (Hong et al., 2023). By coming into close touch with T cells and having paracrine actions, MSCs can control them. Two crucial molecules on MSCs membranes implicated in T-cell regulation are Fas ligand (FASL) and Programmed cell death protein-L1 (PD-L1). By directing the expression of PD-1 or CD80 on T cells, PD-L1 on MSCs prevents T cell growth by sending out inhibitory signals (Luz-Crawford et al., 2012). Similar to this, FALS on MSC’s membrane interacts with T cell’s Fas to cause T-cell apoptosis (Wang L. et al., 2012). Galactagogue-1, a member of the galactagogue family, is extensively expressed on the surface of MSCs and limits T-cell proliferation via controlling inflammation (Sioud, 2011). In addition to these key cell surface molecules, MSCs can also regulate T cells through paracrine effects. Human umbilical cord MSCs have been demonstrated to inhibit T cell activation and development by generating TGF-*β* and preventing T cells from entering the G0 phase Vellasamy et al. (2013). More of the catabolic enzyme indoleamine-2,3-dioxygenase (IDO), which changes tryptophan into kynurenine, may be produced by MSCs. Additionally, they can inhibit T-cell proliferation, activate GCN2(a general control non-suppressible factor), promote local tryptophan depletion, and convert naïve CD4^+^ T cells into Tregs (Munn et al., 2005; Fallarino et al., 2006). In addition, IDO generates soluble compounds like kynurenine and its byproducts that can bind to and activate the aryl hydrocarbon receptor. This increases Tregs development (Mezrich et al., 2010) and causes DCs to take on an immunosuppressive character (Quintana et al., 2010). In addition, it has been demonstrated that MSCs counteract pro-inflammatory and anti-inflammatory responses, prevent T cells from differentiating into the Th1 and Th17, and encourage the development of Tregs (Duffy et al., 2011b; Vellasamy et al., 2013). Furthermore, MSCs modify T-cell polarization and cytokine secretion profiles, transforming pro-inflammatory Th1 cells into anti-inflammatory Th2 cells (Di Nicola et al., 2002; William et al., 2003; Wang et al., 2008). Th1 cytokine production has been shown to be inhibited by MSCs via PGE-2-dependent inhibition, Th17 cytokine production is constrained by MSCs by upregulating PD-1 expression and IL-10 production, and MSCs inhibitory activity is activated through CCL2-dependent inhibition (Darlington et al., 2010; Duffy et al., 2011a; Luz-Crawford et al., 2012; Mohammadzadeh et al., 2014). In addition, it has been found that T-cell apoptosis resulting from inhibition by MSCs enables macrophages to produce TGF-*β*, which promotes the induction of Tregs (Ren et al., 2008). It has been shown that chemokine receptor 2 (CCR2) overexpressing MSCs speed wound healing and encourage the build-up of Tregs in diabetic wounds Kuang et al. (2021). Studies have indicated that Th17 cell development is suppressed by leukemia inhibitory factor (LIF) released by MSCs Cao et al. (2011), and LIF has also been shown to promote the differentiation of Treg cells Spaggiari et al. (2009). In summary, MSCs can function to promote wound healing by regulating T cells. In summary, MSCs can function to promote wound healing by regulating T cells Table 2.

**TABLE 2:** Factors of MSCs in the regulation of T-cell activity.

Factors	Effectiveness	Mechanisms	Reference
PD-L1	Inhibit T-cell proliferation	Interact with PD-1 on T cells	Luz-Crawford et al. (2012)
FASL	Induce T-cell apoptosis	Interact with FAS on T cells	Wang et al. (2012a)
Galectin-1	Downregulate inflammation	Inhibit cytotoxicity of T cells and restore the proliferation of CD4^+^ and CD8^+^ T cells	Sioud (2011)
TGF-*β*	Inhibit T cells activation	Activate TGF-*β* signal pathway and arrest T cells in G0 phase	Vellasamy et al. (2013)
IDO	Inhibit T-cell proliferation and differentiate naive CD4^+^ T cells into Tregs	Increase local tryptophan depletion and activate the stress response kinase general control non-inhibitable factor 2 (GCN2)	Fallarino et al. (2006); Munn et al. (2005)
	Promote Treg cells differentiation	Generate soluble factors (tryptophan metabolites such as kynurenine) that bind and activate aryl hydrocarbon receptor (AHR)	Mezrich et al. (2010)
LIF	Promote Tregs differentiation	Not mentioned	Spaggiari et al. (2009)
LIF	Induce Th17 cells differentiation	Activate ERKs signal and deactivate STAT3 signal	Cao et al. (2011)

### MSCs regulate the activity of other immune cells

Further studies are needed on the regulation of B lymphocyte activity by MSCs. It has been suggested that MSCs inhibit the synthesis of immunoglobulins such as IgG, IgM, and IgA by activated B cells, thus preventing these cells from differentiating into plasma cells. These cells also decrease the expression of chemokines and their receptors at the level of B lymphocytes, which may negatively affect their migratory capacity (Fan et al., 2016; Boukani et al., 2023). A growing number of scholars have investigated the potential of using MSCs to modulate mast cells for wound repair based on their biological properties. Throughout the healing process of wounds, mast cells interact with several other immune cells (Artuc et al., 1999). Mast cells have the ability to produce antimicrobial peptides that prevent skin infections in their early stages (Wang Z. et al., 2012). Mast cells have been shown to produce histamine and VEGF, which cause vascular permeability and permit neutrophil inflow, as well as chymotrypsin and trypsin-like enzymes necessary for the breakdown of extracellular matrix (ECM) (Dvorak, 2005; Younan et al., 2010). Additionally, released histamine promotes the remodeling of the epithelium and the proliferation of keratinocytes (Weller et al., 2006). Trypsin-like enzymes and histamine also encourage the synthesis of collagen and the proliferation of fibroblasts, which enhances wound contraction and is necessary for the proliferative and remodeling phases (Abe et al., 2000; Garbuzenko et al., 2002). Studies have indicated that MSCs significantly decreased inflammation and mast cell degranulation by producing PGE-2 and TGF-*β* (Kashyap et al., 2005; Kim et al., 2015). It was found that media from TNF-*α*-stimulated MSCs reduced mast cell activation and histamine release and attenuated experimental allergic conjunctivitis through a cyclooxygenase-2 (COX-2)-dependent mechanism (Su et al., 2015). More research is required to determine the specific molecular pathways by which human MSCs govern mast cell activity and its consequences for wound healing, although research on animals has shown that MSCs regulate mast cells. Additionally, MSCs inhibit the conversion of immature dendritic cells to adult dendritic cells and limit the movement of dendritic cells into tissues (Maqbool et al., 2019; Boukani et al., 2023). MSCs also can decrease T-cell activation by inhibiting the growth and activation of DCs (Chiesa et al., 2011; Le Blanc and Mougiakakos, 2012). PGE2, secreted by MSCs, can impede DC maturation and antigen-presentation capabilities. It can also lessen T-cell inflammatory processes (Spaggiari et al., 2009). In response to IL-6, PGE-2, and Jagged-2, it has been demonstrated that MSCs may also generate regulatory and tolerogenic DCs, which will suppress T-cell proliferation and stimulate the growth of Tregs (Spaggiari et al., 2009; Zhang et al., 2009; Deng et al., 2014). We believe that the activities of other immune cells should not be ignored during wound healing as well. More research would be necessary to fully comprehend the mechanism behind the interaction between MSCs and other immune cells Table 3.

**TABLE 3:** Factors of MSCs in the regulation of the activity of other immune cells.

Factors	Effectiveness	Mechanisms	Reference
PGE2	Inhibit mast cells degranulation	Act on EP2 and EP4 receptors	Kim et al. (2015)
TGF-*β*	Inhibit the development and maturation of mast cells	Decrease FcvarepsilonRI, c-kit, T1/ST2, and FcgammaR expression, and inhibite granule formation	Kashyap et al. (2005)
IL-6	Enable DC to acquire a tolerant phenotype	Upregulate suppressor of cytokine signaling 1 (SOCS1)	Deng et al. (2014)
PGE2	Inhibit DC differentiation, maturation and functional acquisition	Not mentioned	Spaggiari et al. (2009)

## Pre-implant optimization of MSCs

### The immunomodulatory effects of MSCs are malleable

The immunomodulatory effects of MSCs are not intrinsic but are triggered by inflammation (Wang et al., 2022). Ren et al. (2008) showed that IFN-*γ*, especially in conjunction with TNF-*α* or IL-1*α*/*β* combos, initiates the immunosuppression of T cells by MSCs. This suggests a mutually beneficial interaction between MSCs and the milieux that causes inflammation. Using a checkerboard titration of IFN-*γ* and TNF-*α*, Li et al. (2012) evaluated the impact of cytokine concentration on immunosuppression in MSCs, providing first-hand evidence for the concept that MSCs are malleable in controlling immunological responses, particularly adaptive immunity. The kind and quantity of inflammatory mediators present in the tissue microenvironment determine this particular trait (Moonen et al., 2012; Wang et al., 2014). For instance, IL-17 in the surrounding environment might intensify MSCs’ ability to inhibit the immune system (Han et al., 2014). In contrast, TGF-*β* and IL-10 reduce the immunosuppressive capacity of MSCs (Renner et al., 2009; Xu et al., 2014). Another factor affecting MSCs’ capacity to regulate immunity is their capacity to identify microbial molecular patterns through TLRs. For instance, TLR-3 activation results in anti-inflammatory signaling, while TLR-4 activation causes pro-inflammatory reactions. It’s interesting to note that TLRs have also been linked to significant skin damage healing. According to Lin et al., TLR-4-deficient mice’s wounds produced much less IL-1*β* and IL-6. Mice lacking the TLR-4 gene also showed a substantial decrease in epidermal growth factor at the edges of their wounds (Chen et al., 2013). In order to stimulate the regeneration of hair follicles, Nelson et al. found that TLR-3, together with its downstream effectors, IL-6 and transducer and activator of transcription 3 (STAT3), was activated with the release of dsRNA from wounded skin. In contrast, TLR-3-deficient animals failed to initiate trauma-induced hair regeneration (WIHN), which is detrimental to wound healing (Nelson et al., 2015). Consequently, MSCs can be efficiently enabled by “matching” signals to control inflammatory responses and work in concert with activated MSCs to reestablish tissue homeostasis. Nevertheless, the therapeutic impact of MSCs will be reduced if they come into contact with “mismatched” signals, such as inadequate proinflammatory cytokines and the existence of immunosuppressive substances or factors (Wang et al., 2022). We need to understand the different needs of inflammation regulation in various types of wounds and at other times of wound healing and amplify their “matching signals” to advance the realization of clinical precision therapy. In addition, the potential mechanisms of MSCs’ plasticity in immune regulation will expand the clinical indications of MSCs and guide the development of new therapeutic strategies for MSCs (Wang et al., 2022). This functional flexibility also offers a potential theoretical foundation for optimizing MSCs before transplantation, for example, before therapy, to maximize their ability to promote wound healing.

### Pre-transplantation optimization strategies for MSCs to enhance their immunomodulatory effects

Several studies have shown that modulation of biological, biochemical, and biophysical factors can influence the fate, lineage-specific differentiation, and function of MSCs and enhance their therapeutic potential (Nava et al., 2012; Huang et al., 2015). To increase MSC activity, survival, and therapeutic efficacy, researchers have proposed and summarized a variety of strategies that hold promise for improving clinical outcomes (Baldari et al., 2017; Huerta et al., 2023). In the following, we will discuss in detail several aspects of cytokines, hypoxia, and drug-chemical pretreatment, as well as biomaterial encapsulation and three-dimensional (3D) culture Table 4.

**TABLE 4:** Pre-transplantation optimization strategies for MSCs to enhance their immunomodulatory effects.

Strategies	Factors	Effectiveness	Reference
Cytokine pretreatment	IFN-*γ*	Upregulate IDO, secrete immunomodulatory molecules; upregulate PD-L1 inhibition of T cells effector function; Act on the early phosphorylation of STAT1/STAT3 and the inhibition of mTOR activity	Chinnadurai et al. (2014); De Witte et al. (2016); Vigo et al. (2017)
	TNF-*α*	Upregulate immunomodulatory factors	Prasanna et al. (2010)
	IFN-*γ* and TNF-*α* co-preconditioning	Increase IDO activity and secret high levels of CCL2 and IL-6	François et al. (2012); Zhu et al. (2020)
	IL-17	Inhibit the secretion of Th1 cytokines by T cells and promote the production of suppressor Tregs	Sivanathan et al. (2017), Sivanathan et al. (2015)
Anoxic pretreatment	HIF-1*α*	Impair DCs differentiation, and reduce NK cell-mediated cell lysis	Ejtehadifar et al. (2015)
	miR-146a-5p	Enhance the immunoregulatory effects	Dong et al. (2021)
Drug or chemical pre-treatment	Rapamycin	Reduce mTOR signaling and enhance its immunosuppressive properties *in vitro* through COX-2 and PGE2 upregulation	Wang et al. (2017)
	ATRA	Upregulate COX-2, HIF-1, CCR2, VEGF and other gene expression	Wang et al. (2017)
	H2O2	Promote neovascularization and reduce the local inflammatory response	Bai et al. (2018)
	Rhodiola rosea glycosides	Reverse hyperglycemia-induced inhibition of key wound healing factor expression in bone marrow MSCs	Ariyanti et al. (2019)
Biomaterials package	Hydrogel	Reduce mechanical stress and loss of MSCS during surgery; improve cell differentiation, accelerates normal wound healing	Ansari et al. (2017); Baldari et al. (2017); Chen et al. (2015); Nava et al. (2012); Rustad et al. (2012)
	Chitin nanofiber	Activate the TGF-*β*/smad signaling pathway	Liu et al. (2022b)
	Bioactive Fish Scale Scaffolds	Promote angiogenesis, modulate macrophage polarization to M2 phenotype, and attenuate local inflammatory responses	Lin et al. (2022)
3D Cultivation	TSG6	Work as a countermeasure to TNF-*α* and IL-1 inflammation by negative feedback loop and in particular by inhibiting neutrophil migration	Bartosh et al. (2010); Follin et al. (2016)
	PGE2	Regulate macrophage polarization	Follin et al. (2016)
	HGF	Not mentioned	Follin et al. (2016)

#### Cytokine pretreatment

Numerous studies have shown that the inflammatory state of the receptor should be taken into account before the treatment of MSCs. To increase the efficacy of MSCs-based therapies in clinical settings, it is therefore advised to pretreat MSCs by stimulating them with inflammatory cytokines that mimic the inflammatory microenvironment of tissues before infusion. As a result, MSCs will no longer be able to inhibit the immune system and will secrete more immunomodulatory and anti-inflammatory substances (Song et al., 2020). It has been demonstrated that pretreating MSCs with IFN-*γ* improves their immunosuppressive capabilities. According to Nelson et al., the release of dsRNA from injured skin activates TLR-3 together with its downstream effectors, IL-6 and STAT3. Research indicates that co-cultivating activated lymphocytes and MSCs sensitized to IFN-*γ* can lower the frequency of Th17 cells, secrete more IL-6 and IL-10, and produce less IFN-*γ* and TNF-*α* (Wang et al., 2016). While inhibiting T cell effector activity, IFN-*γ*-sensitized MSCs also upregulate PDL-1, which increases the production of HLA molecules (Chinnadurai et al., 2014). According to the research carried out by Vigo et al. (2017), due to early phosphorylation of STAT1/STAT3 signal transducers and activators of transcription and a decrease in rapamycin-targeting (mTOR) activity, MSCs from mice treated with IFN-*γ* exhibited immunosuppressive characteristics. Genes associated with immunoregulation were upregulated, and genes related to differentiation, proliferation, and stem cells were downregulated as a result of mTOR activity. Inhibiting the mTOR pathway also improves the immunoregulatory potential of human MSCs (Vigo et al., 2017). Chinnadurai et al. (2017) discovered that by activating regulatory molecules (such as IDO), IFN-*γ* pretreatment might restore the immunosuppressive characteristics of senescent MSCs. TNF-*α* pretreatment is frequently utilized in cytokine combinations to precondition MSCs since it also increases the upregulation of immunoregulatory factors in MSCs, albeit to a lesser degree than IFN-*γ* triggering (Prasanna et al., 2010). Co-preconditioning MSCs with IFN-*γ* and TNF-*α*, as shown by Francois et al., boosted IDO activity in MSCs and caused monocytes to differentiate into M2-type macrophages, which were implicated in T-cell suppression, thereby augmenting MSCs’ immunosuppressive capabilities (François et al., 2012). Liu C. et al. (2022) demonstrated that high levels of CCL2 and IL-6 were found in MSCs after co-pretreatment with IFN-*γ* and TNF-*α*, promoting macrophage migration and polarization. Preclinical research has shown that pretreating MSCs with TNF-*α* and IFN-*γ* increased their therapeutic efficiency in the healing of skin wounds (Zhu et al., 2020). Notably, there was a transient expression of human leukocyte antigen DR (HLA-DR) in MSCs when pretreated by combining TNF-*α* with IFN-*γ*, which may lead to unpredictable immune responses and tissue damage (Wen et al., 2021). Therefore, it is essential to monitor HLA-DR levels as one of the critical parameters for quality control to avoid harmful therapeutic effects in MSCs. Furthermore, MSCs treated with IL-17 stimulated the development of suppressive Tregs and prevented T cells from secreting Th1 cytokines (TNF-*α*, IFN-*γ*, and IL-2) (Sivanathan et al., 2017; Wen et al., 2021). According to Ma et al. (2018), MSCs pretreated with IL-17 significantly extended the survival time of allogeneic grafted skins compared to the non-pretreated group. In transplanted skin grafts, they also noticed elevated tagged MSCs levels, a proportion of the Tregs subpopulation, a notable rise in IL-10 and TGF-*β*, and a drop in IFN-*γ* levels. Such preconditioning strategies can mimic the pathological inflammatory environment present in disease states, and the evidence from their practical use points to the significance of inflammation in triggering MSCs’ anti-inflammatory properties (Wang et al., 2014).

#### Anoxic pretreatment

MSCs exist *in vivo* in microenvironments with generally low oxygen tension (i.e.,1%-5% O_2_). To improve MSCs implantation, survival in ischemic microenvironments, and angiogenic potential *in vitro*, moderate hypoxia has been utilized to simulate the survival conditions of MSCs *in vivo*. This has been achieved by significantly changing cellular metabolism during MSCs expansion and enhancing resistance to oxidative stress (Noronha et al., 2019). Numerous investigations have demonstrated that MSCs grown in hypoxic environments release a variety of soluble bioactive substances (De Witte et al., 2016; Saparov et al., 2016) and increase proliferation ability (Kheirandish et al., 2017). HIF-1*α* produced by hypoxia preconditioned MSCs can impair dendritic cell differentiation, reduce NK cell-mediated cytolysis (Ejtehadifar et al., 2015), and increase resident macrophage recruitment to wounds to accelerate wound healing (Mahjoor et al., 2023). Dong et al. (2021) showed that hypoxia pretreatment of MSCs enhanced their immunomodulatory effects and was able to attenuate inflammation by increasing exosomal miR146a-5p.

#### Drug or chemical pre-treatment

Pretreatment of MSCs with drugs or chemical reagents is also a promising strategy to improve the implantation and survival of MSCs in damaged tissues, thus enhancing the therapeutic efficacy (Baldari et al., 2017). The inhibition of the mTOR signaling pathway caused by rapamycin did not affect all MSCs in the same way. Before co-culturing with activated human CD4^+^ T cells or mice splenocytes, BM-MSCs were briefly exposed to rapamycin. *In vitro*, this led to an increase in the immunosuppressive effects of COX-2 and PGE-2, independent of inflammatory stimuli, and a decrease in mTOR signaling. However, after extended rapamycin exposure, MSCs showed no such impact (Wang et al., 2017). Nuclear retinoic acid receptors (RARs) are occupied by all-trans retinoic acid (ATRA), which is another starting component that is used. ATRA is essential for immune system function, differentiation, apoptosis, and cell development. Rat bone marrow MSCs were pretreated with ATRA, increasing the expression of COX-2, hypoxia-inducible factor-1 (HIF-1), CCR2, and VEGF genes. ATRA-sensitized MSCs improved the capacity of wound healing *in vivo* (Wang et al., 2017). Bai et al. (2018) demonstrated that stimulation of adipose MSCs-derived exosomes using low-dose H2O2 promoted flap neovascularization, attenuated flap inflammation, reduced apoptosis, and improved flap survival after ischemia-reperfusion injury. Rhodiola rosea glycoside, the active ingredient in the Tibetan medicine Rhodiola rosea, can regulate the proliferation, migration, and differentiation of MSCs. The pretreatment with Rhodiola rosea glycoside was able to boost the survival rate of MSCs and improve the migratory capacity of MSCs damaged by hyperglycemia. It was also able to successfully reverse the suppression of essential wound healing factors caused by hyperglycemia in bone marrow MSCs. Research conducted *in vivo* revealed that administering Rhodiola rosea glycosides before transplantation considerably boosted MSCs’ ability to promote wound healing in diabetic mice (Ariyanti et al., 2019).

#### Biomaterials package

The development of biocompatible materials has a direct bearing on the use of MSCs in tissue engineering. These materials can enhance MSCs effectiveness by promoting cell survival and directing *in vivo* cell differentiation (Kusuma et al., 2017). Drug delivery, tissue engineering scaffolds, transplantation treatment, and MSCs cell encapsulation are just a few of the applications for hydrogels that have seen widespread use (Akbari et al., 2020). Because of their capacity to replicate ECM, high biocompatibility and flexibility, noninvasive drug administration, and adjustable physical and chemical characteristics, they are the perfect bioactive scaffolding materials for MSCs tissue creation (Shi et al., 2018b). The viscosity of hydrogels reduces the mechanical forces exerted in syringe-based drug delivery, significantly reducing the loss of MSCs during the procedure (Baldari et al., 2017). In addition, encapsulation/culture of MSCs in hydrogels improved cell differentiation, accelerated routine wound healing, and promoted neovascularization (Rustad et al., 2012; Chen et al., 2015), cell viability, homing, and proliferation (Nava et al., 2012; Ansari et al., 2017). Because of their porous nature, chitin nanofibers are an excellent material for wound dressings since they match the fibrous structure of the extracellular matrix seen in nature. It also encourages cell proliferation, permits the diffusion of nutrients, oxygen, and waste products, and absorbs secretions from wounds to prevent bacterial infections and excessive dehydration. According to Liu Y. et al. (2022), MSCs-loaded *β*-chitin nanofiber hydrogels caused a significant increase in angiogenesis, which in turn encouraged the healing and repair of damaged tissue. Furthermore, they observed that these hydrogels decreased Smad phosphorylation while increasing the synthesis of VEGF, *α*-SMA (*α*-smooth muscle actin), and matrix metalloproteinase tissue inhibitory factor-1 (TIMP-1). Using a decellularization and decalcification procedure, bioactive fish scale scaffolds were produced. This allowed for the preservation of residual bioactive substances within the fish scale scaffolds, and the surface of the decalcified fish scale retained oriented collagen nanofibers, which facilitated the growth of oriented cells in a suitable environment. Lin et al. (2022) found that the fish scale scaffolds loaded with MSCs effectively promoted angiogenesis, regulated macrophage polarization toward the M2 phenotype polarization, and attenuated local inflammatory responses.

#### 3D cultivation

The physical microenvironment influences the function of MSCs. Cell polarity and cell-to-cell contacts are crucial, as demonstrated by studies employing cell aggregates Kusuma et al. (2017). By simulating *in vivo* developmental and signaling activity, 3D cultivation, often called spherical cultivation, increases cell-to-cell and cell-ECM interactions, enhancing the therapeutic potential of human MSCs (Kouroupis and Correa, 2021). The phenotypic of cultured cells, including proliferation, cell-cell interactions, and paracrine factor gene expression, can be changed by 3D culture in comparison to traditional 2D culture (Ryu et al., 2019; Białkowska et al., 2020). Sphere formation increased the survival of MSCs after transplantation (Bhang et al., 2012), enhanced tissue regeneration properties, reduced *in vitro* senescence, and, more importantly, enhanced angiogenesis and anti-inflammatory effects (Cesarz and Tamama, 2016). Hildebrandt et al. showed improved nutrient delivery and enhanced viability of MSCs based on rotary cultures, suspension-droplet technique, and 3D cultures in non-adherent culture vessels (Hildebrandt et al., 2011). Bartlett et al. showed improved nutrient delivery and enhanced viability of MSCs (Hildebrandt et al., 2011). It was demonstrated by Bartosh et al. (2010) that the high expression of TSG-6 was responsible for the powerful anti-inflammatory effects of 3D-cultured MSCs. It was demonstrated that via increasing PGE-2 and HGF levels, 3D-cultured MSCs also strengthened immunosuppressive effects (Follin et al., 2016). Many techniques, including pellet culture, suspension drops, spinning flasks, magnetic levitation, and space culture, have been used to create spheroids thus far (Blaber et al., 2014; Ryu et al., 2019; Wnorowski et al., 2019; Białkowska et al., 2020). For the most part, sphere creation is a labor-intensive procedure that takes a long time. Human MSCs were cultured by Park et al. (2023) using acoustic levitation to produce spheroids, which took significantly less time, had a denser and more homogeneous morphology, and showed higher levels of metabolic activity, viability, and proliferative capacity. The genes encoding angiogenic, senescent, and proliferative factors were also expressed at elevated levels.

## Conclusion

Stem cells exhibit the capacity to repair tissue and heal wounds. Studies in both the clinic and the laboratory have shown that MSCs-based inflammatory regulation treatment is a successful tactic for accelerating wound healing. Nonetheless, the primary issue limiting their practical use is the limited survival and retention of cells following implantation. Current research emphasizes how critical it is to control inflammation early in the healing process and how MSCs can help heal wounds by influencing the local inflammatory milieu through primary immunomodulatory action. Furthermore, the adaptability of MSCs’ immunomodulation is highlighted, and methods to boost MSCs’ therapeutic effectiveness—which has been demonstrated to raise MSCs migration and survival, encourage angiogenic potential, improve immunomodulation, and increase overall efficacy—are further explored. These findings hold potential for the advancement of MSCs-based treatment approaches. In the clinical management of wounds, clinicians tend to administer antimicrobial and anti-inflammatory treatments immediately after trauma to prevent excessive inflammatory responses. In contrast, the need for inflammation may vary during different periods of wound healing. Thus, to advance the realization of precision therapy in the clinic, more thorough research is still needed to understand the molecular mechanisms underlying the interactions between MSCs and each type of immune cell, particularly the distinct requirements for inflammation regulation for different kinds of wounds and varying periods of wound healing. Future research endeavors may expand upon this study and offer valuable perspectives on the practical applications of these results.
